# Potential of Lichen Compounds as Antidiabetic Agents with Antioxidative Properties: A Review

**DOI:** 10.1155/2017/2079697

**Published:** 2017-04-12

**Authors:** Vinitha M. Thadhani, Veranja Karunaratne

**Affiliations:** ^1^Sri Lankan Institute of Nanotechnology, Mahenwatta, Pitipana, Homagama, Sri Lanka; ^2^Department of Chemistry, University of Peradeniya, Peradeniya, Sri Lanka

## Abstract

The advancement in the knowledge of potent antioxidants has uncovered the way for greater insight in the treatment of diabetic complications. Lichens are a rich resource of novel bioactive compounds and their antioxidant potential is well documented. Herein we review the antidiabetic potential of lichens which have received considerable attention, in the recent past. We have correlated the antidiabetic and the antioxidant potential of lichen compounds. The study shows a good accordance between antioxidant and antidiabetic activity of lichens and points out the need to look into gathering the scarce and scattered data on biological activities for effective utilization. The review establishes that the lichen extracts, especially of* Parmotrema *sp. and* Ramalina* sp. have shown promising potential in both antidiabetic and antioxidant assays. Ubiquitous compounds, namely, zeorin, methylorsellinate, methyl-*β*-orcinol carboxylate, methyl haematommate, lecanoric acid, salazinic acid, sekikaic acid, usnic acid, gyrophoric acid, and lobaric acid have shown promising potential in both antidiabetic as well as antioxidant assays highlighting their potential for effective treatment of diabetic mellitus and its associated complications. The available compilation of this data provides the future perspectives and highlight the need for further studies of this potent herbal source to harvest more beneficial therapeutic antidiabetic drugs.

## 1. Introduction

Diabetes mellitus (DM) is an ever increasing global epidemic and one of the most challenging health problems of 21st century. In 2010, more than 285 million people around the world were afflicted with diabetes, and it was then estimated that the number of people with diabetes will increase to 439 million by 2030. Interestingly, the reports of 2015 show that globally 415 million (215.2 million men and 199.5 million women) had DM with a prevalence of 8.8%. In other words, one in eleven people have DM and global expenditure for treating it in 2015 alone was US$ 673 billion (12% of health expenditure) [1].

Two main groups of DM are distinguished: (1) autoimmune T1DM or insulin dependent DM or juvenile DM and (2) T2DM or noninsulin dependent DM or Maturity Onset DM. About 90% of people with DM around the world have type 2 DM (T2DM) [2].

In T1DM, *β*-cells in the pancreas are destroyed and do not secrete adequate insulin; treatment of T1DM requires insulin replacement via injection. T2DM is characterized by insulin resistance and a diminished capacity for insulin secretion by *β*-cells of the pancreas. T2DM is considerably more amenable to therapeutic drug intervention and is treated with insulin sensitizers, or through methods which reduce the plasma glucose levels. Natural products and herbal medicines that have claimed to be efficacious in the treatment of DM are thus most efficient in the treatment of T2DM [3].

Recent basic and clinical studies have exposed new understandings into the role of antioxidants to combat diabetic complications [4]. Oxidative stress plays a significant part in the pathogenesis of diabetes and its ramifications as it leads to the dysfunction of *β*-cells. Antioxidants on the other hand protect *β*-cells from apoptosis and preserve their function [5]. Therefore, if a compound shows good antioxidant activity, it is anticipated that it would show greater effects on diabetes and its complications as well. Thus antioxidant therapy recommends a different, innovative, and fundamental approach towards diabetes treatment [6, 7].

Lichens are composite organisms consisting of a symbiotic association between a fungal partner (mycobiont) and one or more photosynthetic partners (photobiont) usually either green algae or cyanobacterium or both. Lichens are found in all ecosystems, including the most extreme environments on earth-arctic tundra, hot deserts, icebergs, rocky coast, toxic heaps, and so on. Lichens produce characteristic and unique substances which may help them to survive in these extreme environments [8]. Around 1050 lichen metabolites are known up to date [9]. Importantly, the last decade witnessed renewed and growing interest in lichen substances as a source of novel, pharmacologically active biomolecules [10, 11]. Overall, tropical lichens are one of the least studied cryptogams. For example, in Sri Lanka, new species and new records of lichens are being discovered at a rapid rate and in the coming years the number of tropical lichens recorded will contribute to new knowledge of their pharmaceutical potential [12, 13]. Interestingly, wide array of biological activities have been reported revealing the pharmaceutical importance of Sri Lankan lichens [14–23].

Adequate literature exists, for certain lichens to be a viable source of antioxidants [9, 10, 24, 25]. Their antioxidant potential has been assessed in a number of assays, including, DPPH radical scavenging, reducing power, superoxide anion radical scavenging, nitric oxide radical scavenging, and lipid peroxidation inhibition. Antioxidant properties of 75 lichen species and 65 isolated metabolites were reviewed and reported [25]. Further, over 66 lichen extracts have been reviewed and analyzed for their antioxidant potential. [26]. However, compared to crude extracts, a limited number of publications exists for the antioxidant activity of pure compounds. The antioxidant activity of number of lichen compounds with its structure activity relationship has been reported [18, 19].

Further confounding the available information, compared to the antioxidant potential, limited information exists on evaluation of the efficacy of lichens as antidiabetic agents. A number of different approaches have been used, including *α*-amylase inhibitory [27–34], *α*-glucosidase [35, 36], and PT1B inhibitory activity [37–40], antiglycation [41, 42], along with a few in vivo studies to identify the potential of lichens in treating DM [43–45].

Importantly, most of the extracts and pure compounds of lichens reported for its antidiabetic potential have been separately studied for their antioxidant potential. Here, we summarize the antidiabetic effect of lichens by referring to recent studies, including those reported by us with the perspective of how their reported radical scavenging activities would influence the relationship (if any) between the antioxidant potential and the antidiabetic activities.

## 2. In Vitro Antidiabetic Activities of Lichen Extracts

Several studies have discovered the positive potential of exploring lichens as potent antidiabetic agents. Their hypoglycemic action has been assessed in different methods, including through their inhibitory activity of carbohydrate hydrolyzing enzymes (*α*-amylase and *α*-glucosidase) or protein tyrosine phosphatase 1B (PTP1B), which is recognized as the major negative regulator in insulin signaling, or through antioxidative effect, involved in restoring of insulin secreting pancreatic cells [27].

This review summarizes the reported antidiabetic activity of lichens using *α*-amylase, *α*-glucosidase, antiglycation, and protein tyrosine phosphatase 1B (PTP1B) inhibitory assays as well of some work reported by us.

### 2.1. *α*-Amylase Activity of Lichen Extracts

*α*-Amylase is the key enzyme involved in carbohydrate digestion. It hydrolyzes starch and glycogen into maltose and ultimately increases the blood sugar.

Several lichen extracts have been evaluated for *α*-amylase inhibitory activity and have shown beneficial effects in bringing down the pace of digestion and assimilation of sugars and thereby leading to the effective management of type 2 diabetes by decreasing the postprandial hyperglycemia [28].

Up to now, 22 lichen extracts including some in more than one study have been assessed and shown a positive potential in the *α*-amylase inhibitory assay. These include* Everniastrum cirrhatum*,* Usnea sinensis*,* Ramalina conduplicans*,* R. hossei*,* Parmotrema tinctorum, P. pseudotinctorum,* [29],* Flavoparmelia caperata*,* Physcia aipolia*,* Heterodermia leucomela* [30],* Ramalina sinensis*,* Heterodermia leucomela*,* Herpothallon *sp.,* Parmotrema reticulatum *[31],* Parmotrema tinctorum *[32],* Usnea articulate*,* Ramalina pollinaria*,* Ramalina hyrcana*,* Cladonia rei*,* Flavoparmelia caperata*,* Parmotrema chinense*,* Punctelia subrudecta*,* Punctelia borreri*,* Hyperphyscia adglutinata*, and* Peltigera praetextata *[33].

Wider interest has been received by plant natural polyphenols for their *α*-amylase inhibitory activity [46]. Nevertheless, no reports exist for the evaluation of pure lichen compounds against the *α*-amylase. However, it could be resolved that the *α-*amylase inhibitory activity shown by above lichens could be attributed to their phenolic compounds.

The structure activity relationship of polyphenols isolated from other plant sources has shown that *α-*amylase inhibitory activity is influenced by a number of hydroxyl groups and their positions, methylation, methoxylation, glycosylation, and so on. Broadly, it is considered that hydroxylation of phenols increases the *α-*amylase inhibitory activity and methoxylation, blocks the free hydroxyl groups, and reduces the inhibitory activity [47].

Molecular docking studies have revealed that, overall, the inhibitory activity of phenols depends on two parameters: (i) hydrogen bonding capacity of the OH groups of the phenols with the side chains of amino acids such as Asp197, and Glu233 and (ii) planarity of aromatic rings to form an efficient conjugated *π*-*π* system with the indole Trp59 of the active site [48].

It is important to understand that lichen polyphenols are structurally distinct from other phenols such as flavonoids, catechins, and tannis found in higher plants. Lichen phenolics are mainly monocyclic phenols, depsides, depsidones, dibenzofurans, derived through the acetyl-polymalonly pathway, with mainly orsellinic acid as the basic unit in the biosynthesis. Thus, it would be interesting to estimate the *α-*amylase inhibitory activity of these different classes of polyphenols and to analyze its structure activity relationship, to describe its mechanism of action.

### 2.2. *α*-Glucosidase Inhibitory Activity of Lichen Compounds

*α*-Glucosidase is an another key enzyme involved in the digestion of dietary carbohydrates in humans. It hydrolyzes oligosaccharides and disaccharides into glucose, which is absorbed through the gut wall to become blood glucose. Thus, inhibition of *α*-glucosidase activity is viewed as one of the most effective therapeutic approaches in the reduction of glucose levels in plasma and, as a consequence, the suppression of postprandial hyperglycemia.

However, compared to *α-amylase* inhibitory activity, an extensive literature survey showed only four lichen, namely,* Caloplaca biatorina* [34],* Ramalina celastri, R. nervulosa, and R. pacifica, *[36] where extracts were evaluated, for their *α*-glucosidase inhibitory activity. On the other hand, 6 common secondary metabolites have been evaluated in two different studies and have shown promising antihyperglycemic effect. These include zeorin, methyl-*β-*orcinolcarboxylate, methylorsellinate [16, 35], sekikaic acid, salazinic acid, and dibenzofuran usnic acid [36].

Monocyclic aromatics, methyl-*β-*orcinolcarboxylate, and methylorsellinate exhibited 4-5-fold higher activity than acarbose [16], whereas depsidone salazinic acid, depside sekikaic acid, and dibenzofuran usnic acid showed compatible IC_50_ value as compared to the standard acarbose [36]. The kinetic inhibition studies of salazinic acid, sekikaic acid, and usnic acid towards *α*-glucosidase enzyme revealed the competitive type of suppression by both salazinic acid and sekikaic acid and noncompetitive inhibition by usnic acid [36]. The kinetic studies of methyl-*β-*orcinolcarboxylate and methylorsellinate are not reported.

Several polyphenols isolated from different sources, especially flavonoids, have been extensively reviewed as inhibitors of *α*-glucosidase. Detailed SAR has revealed that both *α*-amylase and *α*-glucosidase share the same properties in terms of structural requirements for inhibition [47, 48]. However, similar to *α*-amylase no reports exist on SAR of lichen polyphenols against *α*-glucosidase. Thus, it would be vital to carry out docking based studies to understand which type(s) of hydroxyl moieties undergo H bonding with active sites amino acid residues.

Interestingly, the ubiquitous triterpenoid zeorin found exclusively in almost all lichens, possessed the most significant *α*-glucosidase inhibitory activity with an IC_50_ value of 100.0 ± 0.3 *μ*M, when compared to standard drugs, acarbose (IC_50_ = 700.0 ± 10.4 *μ*M), and 1-deoxynojirimycin (IC_50_ = 425.0 ± 8.9 *μ*M) [16].

The role of triterpenoids in the management of diabetic mellitus and its complications has received as much attention as plant polyphenols. Pentacyclic triterpenoids, belonging to oleanane, ursane, and lupane types, isolated from different plant sources, have been extensively reviewed as *α*-glucosidase inhibitors [49]. On the other hand, zeorin is structurally different from any of the above pentacyclic triterpenoids and it would be noteworthy to study its mode of action.

### 2.3. Protein Tyrosine Phosphatase Inhibitory Assay

Protein tyrosine phosphatase 1B (PTP1B) has been recognized as a major negative regulator of insulin signaling and therefore has been identified as a possible drug target for the treatment of type 2 diabetes and obesity. Prior to studies by Seo et al., in 2009, no reports existed on PTP1B inhibitory activity of lichens [37]. This group has gone on to study further, several lichen extracts as well as their isolated secondary metabolites [37–40] along with their kinetic studies, to draw considerable attention to the evaluation of different lichens against PTP1B inhibitory activity.

The Antarctic lichens which were evaluated against PTP1B inhibitory activity were* Umbilicaria antarctica*,* Stereocaulon alpinum* [37, 38],* Lecidella carpathica* [39], and* Huea *sp. [40]. The compounds which resulted in the above PTP1B inhibitory activity were identified as triterpenoid, zeorin (hopane-6*α*,22-diol), monocyclic aromatic compounds as methyl-*β*-orcinol carboxylate (atraric acid), methylorsellinate, methyl haematommate, depsides lecanoric acid, gyrophoric acid, atranorin, brialmontin 1, and depsidone lobaric acid along with four new diterpene furanoids. Gyrophoric acid consisting of three orsellenic acid units had shown almost 9 times more potent PTP1B inhibitory activity as compared to lecanoric acid which consisted of two orsellenic rings. Similarly, lecanoric acid had shown almost 9 times more potent inhibitory activity than monocyclic methylorsellinate. On the other hand, Brialmontin 1, with more hydrophobicity when compared to lecanoric acid, has shown higher inhibitory potential. These results reveal that inhibitory potency appears to get stronger with increase in lipophilicity. Zeorin, the most lipophilic compound out of the tested compounds, had shown the highest potential as PTP1B inhibitor.

Further, over 27 triterpenoids of oleanane, ursane, and lupane types, isolated from different sources, have been reported as PTP1B inhibitors [50]. However, zeorin was the most potent amongst the reported PTP1B inhibitors and inhibited PTP1B in a competitive manner. Additionally, zeorin displayed selectivity towards PTP1B over other PTPs, such as TCPTP (T-cell protein tyrosine phosphatase). Structurally zeorin is different from other triterpenoids isolated from plant sources. It lacks a carboxyl group, which is considered as an essential feature, related to the inhibitory activity. Also zeorin was the only triterpenoid, amongst the PTP1B active triterpenoids, which lacked the C-3 hydroxyl group, another essential feature related to inhibitory activity. It would be interesting to see the mode of action of zeorin, which has a C-6 hydroxyl group.

### 2.4. Antiglycation Activity of Lichen Compounds

Increased glycation and buildup of advanced glycation end products have been implicated in diabetes complications. Thus, there is considerable interest in antiglycation compounds because of their therapeutic potential against diabetes.

Literature on crude lichen extracts for antiglycation was not found; however, antiglycation activity of several secondary metabolites has been described [41]. Amongst the compounds examined, the depside atranorin had shown substantial activity as an antiglycation agent along with divaricatic acid and usnic acid. In an another study, ethyl haematommate, ethyl orsellinate, lecanoric acid, and gyrophoric acid had shown antiglycation activity [42].

Table 1 summarizes the reported antidiabetic lichens and compounds along with their tested activities.

Interestingly, ubiquitous compounds, namely, zeorin, methylorsellinate, methyl-*β*-orcinol carboxylate, atranorin, and lecanoric acid have shown antidiabetic activity in more than one assay including in *α*-glucosidase, PTP1B, and antiglycation activities, revealing the multidiabetic benefits of these lichen compounds.

### 2.5. In Vivo Antidiabetic Studies

The in vivo antidiabetic studies of three lichen extracts, namely,* Cladonia humilis *[43],* Parmotrema grayana* [44] and* P. hababianum* [45] have been described to prove the in vivo antidiabetic potential of lichens.

Ethanolic extracts of* P. hababianum*, which had shown potent in vitro antioxidant activity, had also proven to be antihyperglycemic when tested against streptozocin induced diabetic rats [45]. Herein we report the hypoglycemic effect of authentic zeorin on streptozocin induced diabetic rats. Results revealed that zeorin at 50 mg/kg was able to bring down the blood glucose level from 500 mg/dL to 400 mg/dL within 2 hours, whereas zeorin, at 100 mg/Kg, reduced the glucose level from 580 mg/dL to 380 mg/dL. Acarbose at 5 mg/Kg reduced the serum glucose from 520 mg/dL to 380 mg/dL.

## 3. Antioxidant Activities of Lichen Compounds

Lichens appear to be a promising source of unique phenolic compounds, which do not occur in higher plants, and other free living fungi. The antioxidant properties of these phenolic compounds, as well as their crude extracts, have been thoroughly assessed using both in vitro and in vivo studies. Ample data exist to prove lichens as a reliable source of antioxidants. There are already several reviews on antioxidant activities of lichen extracts and their compounds [25, 26]. Reviewing the antioxidant activities of lichen compounds is beyond the scope of this study. Herein we capture the antioxidant potential of only the lichens and their compounds which have been reported as antihyperglycemic agents, to further reveal their multidiabetic potentials.

**Figure 1:**
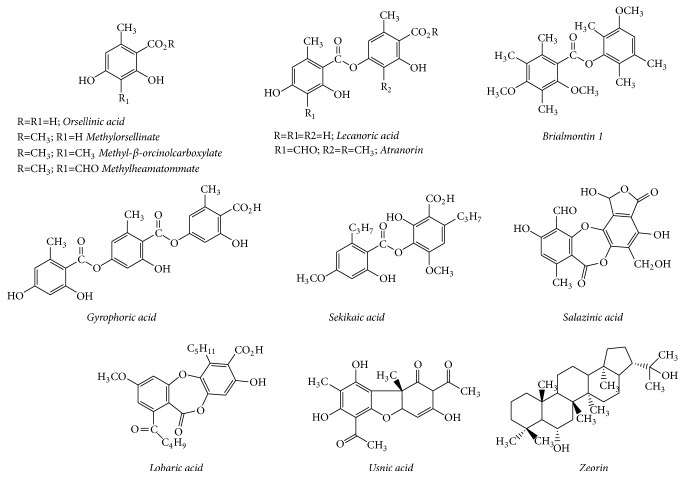
Secondary metabolites of lichens reported as both antioxidant and antidiabetic.

The results are summarized in Table 2.  Figure 1 provides the structures of the compounds which were active in both antidiabetic and antioxidant assays.

Interestingly, most of these lichen extracts and lichen compounds which are reported as antihyperglycemic (Table 1) have been separately studied and reported as antioxidants as well.

It is encouraging to note that out of the 22 lichens extracts which are reported for their antidiabetic potential, 19 have shown antioxidant activity (Table 2). The lichen extracts, namely,* Flavoparmelia caperata, Parmotrema reticulatum, P. tinctorum, P. pseudotinctorum, P. chinense, Everniastrum cirrhatum, Usnea sinensis, U. articulate, Ramalina pollinaria, R. celastri, R. nervulosa, R. pacifica, R. conduplicans, R. hossei, Punctelia subrudecta, Peltigera praetextata, Umbilicaria antarctica, Stereocaulon alpinum,* and* Caloplaca biatorina *are reported for both their antihyperglycemic and promising antioxidant activity.

Similar observations were made in the case of pure compounds. Of the 17 known secondary metabolites which have shown antidiabetic activity, namely, zeorin, methylorsellinate, methyl-*β*-orcinol carboxylate, methyl haematommate, orsellinic acid, lecanoric acid, atranorin, sekikaic acid, salazinic acid, gyrophoric acid, usnic acid, lobaric acid, divaricatic acid, gyrophoric acid, ethyl haematomate, ethyl orsellinate, Brialmontin 1 (Table 1), 13 are already reported for their antioxidant potentials (Table 2).

Importantly, methylorsellinate, methyl-*β*-orcinol carboxylate, and lecanoric acid, which had shown promising antidiabetic properties in more than one assay (Table 1), are also reported as antioxidants in diverse assays (Table 2). The other compounds such as salazinic acid, gyrophoric acid, sekikaic acid, usnic acid, methyl haematommate, orsellinic acid, lobaric acid, and divaricatic acid have also been reported for both antidiabetic and antioxidant potentials. Positive action in both assays makes these phenolic metabolites promising sources to be measured for their effects in the treatment of diabetes mellitus as well as its related ramifications.

Antioxidant activity of ethyl haematommate, ethyl orsellinate, and brialmontin 1 is not reported, whereas zeorin has not shown potent antioxidant activities in DPPH, SOI, NO, and metal chelating assays [18].

## 4. Pharmaceutical Potential of Lichen Compounds as Antidiabetic Agents

Diverse antidiabetic benefits of lichen compounds could be summarized as inhibition of starch digestion by inhibition of digestion enzymes (*α*-amylase, *α*-glucosidase), PTP1B inhibitory activity, inhibition of advanced glycation end products, and antioxidants activities, resulting in protection of *β*-cells from apoptosis.

Further, methylorsellinate, methyl-*β*-orcinol carboxylate, methyl haematommate, orsellinic acid, and lobaric acid have likewise been reported as immunomodulators in addition to antioxidants and antiglycation agents [22]. The immunomodulatory agents are being used as adjuvant therapy in oxidative stress induced diseases to ameliorate the immune system. Thus, these compounds also show a huge potential to be pharmaceutically exploited.

Interestingly, most of the above bioactive compounds are ubiquitous compounds and it would be beneficial to develop novel techniques for direct identification of these compounds in a given extract, rather than the laborious and hectic processes of isolation, purification, and structure elucidation. A recent report on “Rapid identification of lichen compounds based on the structure–fragmentation relationship using ESI-MS/MS analysis” [75] may have paved the way for such analysis.

However, there is a need for more precise investigations to examine the clinical value of both isolated pure compounds and crude extracts and to elucidate their mechanisms of action. Apart from clinical validation and elucidation of their mechanism of action, biosafety studies of the compounds are also important to legitimately use the potential bioactive compounds for the further development of future lead drugs.

## 5. Conclusion

Lichen metabolites have demonstrated promising results as a reservoir of biological active compounds. Even though the studies on antioxidant activities of lichens have a comparatively long prior history, the reports on the potential of lichens as antidiabetic agents have evolved in the very recent past. Even from the limited data, the diverse diabetic potential is signified. Several lichens extracts have shown promising effects both in the antioxidant and in the antidiabetic assays. Interestingly and importantly, out of the 22 lichens extracts studied for their antidiabetic potential, 19 have already been established as antioxidants in separate studies. Likewise, of the 17 known secondary metabolites which have proven antidiabetic activity, 13 are recognized as antioxidants in various assays. Thus the study shows that there is a good accordance between antioxidant and antidiabetic activity of lichens.

This review points out the importance of studying lichen specific, polyphenols as *α*-amylase and *α*-glucosidase inhibitors, to understand their mode(s) of action. Further this review highlights that even though there are substantial data on the plant derived triterpenoids such as oleanolic acid, as antidiabetic agents, lack of data on the more potent triterpenoid zeorin limits its potential application.

A detailed study of the potential protective role of these agents needs to be carried out to exploit their potential for the effective treatment of DM and associated complications.

Even from the limited number of studies it can be concluded that lichen-derived bioactive compounds hold great promise for biopharmaceutical applications as reported for antidiabetic activity and also antioxidant properties and point out the need to look into gathering the scarce and scattered data on biological activities for effective utilization.

However, unfortunately, lichens have been essentially overlooked to a great extent by the modern pharmaceutical industry, despite all the evidence of biological activity in lichen extracts provided in literature.

## Figures and Tables

**Table 1:** Reported lichens and their compounds as antidiabetic agents.

Lichens	Compounds	Activity	Author [Ref.]
*Flavoparmelia caperata* *Physcia aipolia* *Heterodermia leucomela*	—	*α*-Amylase	Shivanna et al., 2015 [30]

*Ramalina sinensis* *Heterodermia leucomelos* *Herpothallon sp*. *Parmotrema reticulatum*	—	*α*-Amylase	Hengameh et al., 2016 [31]

*Everniastrum cirrhatum* *Usnea sinensis* *Ramalina conduplicans* *Ramalina hossei* *Parmotrema tinctorum* *P. pseudotinctorum*	—	*α*-Amylase	Vinayaka et al., 2013 [29]

*Usnea articulate* *Ramalina pollinaria* *Ramalina hyrcana* *Cladonia rei* *Flavoparmelia caperata* *Parmotrema chinense* *Punctelia subrudecta* *Punctelia borreri* *Hyperphyscia adglutinata* *Peltigera praetextata*	—	*α*-Amylase	Valadbeigi and Shaddel, 2016 [33]

*Caloplaca biatorina*	—	*α*-Glucosidase antioxidant	Valadbeigi, 2016 [34]

*Ramalina celastri *	Salazinic acid,	*α*-Glucosidase	
*R. nervulosa *	Sekikaic acid,		Verma et al., 2012 [36]
*R. pacifica*	Usnic acid	Antioxidant	

*Cladonia* sp.	Zeorin, Methyl-*β*-orcinol carboxylate Methylorsellinate	*α*-Glucosidase	Thadhani et al., 2011 [35] Karunaratne et al. 2014 [16]

*Umbilicaria antarctica* *Stereocaulon alpinum*	Gyrophoric acid, Lecanoric acid, Methyl orsellinate	PTP1B	Seo et al., 2009 [37]

*Stereocaulon alpinum*	Lobaric acid, Pseudodepsidones	PTP1B	Seo et al., 2009 [38]

*Lecidella carpathica*	Zeorin, Methyl-*β*-orcinol carboxylate, Brialmontin 1, Atranorin, Methylhaematomate	PTP1B	Seo et al., 2011 [39]

*Huea* sp.	Diterpene furanoids	PTP1B	Cui et al., 2012 [40]

*Parmotrema grayana*	Atranorin Divaricatic acid Usnic acid	Antiglycation	Thadhani 2013 [41]

*Parmotrema cooperi*	Lecanoric acid Gyrophoric acid Ethyl haematomate Ethyl orsellinate Orsellinic acid	Antiglycation	Choudhary et al., 2011 [42]

**Table 2:** Antioxidant potential of lichens/compounds reported as antidiabetic.

Lichen extracts/compounds	Antioxidant activity [References]	Assayed bioactivity type
*Flavoparmelia caperata *	Stojanović et al., 2010 [51] Mitrović et al., 2011 [52]	DPPH & FRAP assays DPPH

*Parmotrema reticulatum*	Ghate et al., 2013 [53] Sharma, 2012 [54] Rajan et al., 2016 [55]	HORAC, ORAC, DPPH, SOI, & NOS DPPH & FRAP assays DPPH

*Parmotrema tinctorum*	Raj et al., 2014 [32] Vivek et al., 2014 [56] Rajan et al., 2016 [55]	DPPH, ABTS, SOI, HORAC DPPH DPPH

*Parmotrema*	Rajan et al., 2016 [55]	DPPH
*Pseudotinctorum*	Kumar et al., 2010 [57]	DPPH & FRAP
*Parmotrema chinense*	Vivek et al., 2014 [56]	DPPH

*Everniastrum cirrhatum*	Kekuda et al., 2011 [58] Kumar et al., 2014 [59]	DPPH, FRAP & MC DPPH & FRAP

*Usnea sinensis*	Prateeksha et al., 2016 [60] Devahat et al., 2007 [61]	DPPH

*Ramalina pollinaria*	Gulluce et al., 2006 [62]	DPPH & ALP

*Ramalina celastri *	Verma et al., 2012 [36]	DPPH, ALP, SOI, NOS & TEAC

*R. nervulosa*	Verma et al., 2012 [36]	DPPH, ALP, SOI, NOS & TEAC

*R. pacifica*	Verma et al., 2012 [36]	DPPH, ALP, SOI, NOS & TEAC

*Ramalina conduplicans*	Luo et al., 2010 [63] Xia et al., 2015 [64] Kumar et al., 2009 [65]	DPPH, FRAP & ALP DPPH & ABTS DPPH

*Ramalina hossei*	Kumar et al., 2009 [65] Ranković, 2015 [66]	DPPH DPPH

*Punctelia subrudecta*	Mastan et al., 2014, [67]	DPPH and HORAC
*Peltigera praetextata*	Zambare and Christopher 2012 [10]	

*Umbilicaria antarctica*	Luo et al., 2009 [68]	DPPH, SOI, ALP
	Strzalka et al., 2011 [69]	tocopherols, plastoquinone & plastochromanol

*Stereocaulon alpinum*	Bhattarai et al., 2008 [70] Bhattarai et al., 2013 [71]	DPPH DPPH
*Caloplaca biatorina*	Valadbeigi; 2016 [34]	DPPH, FRAP

Salazinic acid	Selvaraj et al.; 2015 [72]	DPPH, FRAP, MC, HORAC, ALP, phosphomolybdenum SOI
	Manojlovic et al., 2012 [73]	DPPH, SOI
Gyrophoric acid	Kosanic et al., 2014 [74]	DPPH, SOI, FRAP

Sekikaic acid, Usnic acid Methyl-*β*-orcinol carboxylate Methylorsellinate Lecanoric acid Methylhaematomate Orsellinic acid, Lobaric acid, Divaricatic acid	Thadhani et al., 2011 [18]	DPPH, SOI, NOS & MC

DPPH: (1, 1diphenyl-2-picrylhydrazyl) radical scavenging method, FRAP: ferric reducing antioxidant power, MC: metal chelating, SOI: super oxide inhibitory, ALP: anti-linoleic acid peroxidation assay, NOS: nitric oxide-scavenging assay; TEAC: trolox equivalent antioxidant capacity assay; HORAC: hydroxyl radical antioxidant capacity, and ORAC: oxygen radical antioxidant capacity.
